# Measurement properties of the 12-item Short Form Health Survey version 2 in Australians with lung cancer: a Rasch analysis

**DOI:** 10.1186/s12955-021-01794-w

**Published:** 2021-05-31

**Authors:** Sze-Ee Soh, Renata Morello, Darshini Ayton, Susannah Ahern, Ri Scarborough, Claire Zammit, Margaret Brand, Robert G. Stirling, John Zalcberg

**Affiliations:** 1 Department of Physiotherapy, Monash University, Melbourne, VIC Australia; 2 Department of Epidemiology and Preventive Medicine, Monash University, Melbourne, VIC Australia; 3 Department of Respiratory Medicine, The Alfred Hospital, Melbourne, VIC Australia; 4 Department of Medicine, Monash University, Melbourne, VIC Australia

**Keywords:** Rasch analysis, Health status, Lung cancer, Psychometrics

## Abstract

**Background:**

The 12-item Short-Form Health Survey version 2 (SF-12v2), a widely used, generic patient-reported measure of health status that provides summary scores of physical and mental health. No study to date has examined the measurement properties of the SF-12v2 in patients with lung cancer using Rasch analysis. The aim of this study was to extend the psychometric evaluations of the SF-12 within the lung cancer population to ensure its validity and reliability to assess the health status in this population.

**Methods:**

Participants in the Victorian Lung Cancer Registry (VLCR) who completed the SF-12v2 between 2012 and 2016 were included in this study. The structural validity of the SF-12v2 was assessed using Rasch analysis. Overall fit to the Rasch measurement model was examined as well as five key measurement properties: uni-dimensionality, response thresholds, internal consistency, measurement invariance and targeting.

**Results:**

A total of 342 participants completed the SF-12v2 three months following their lung cancer diagnosis. The SF-12 Physical Component Score (PCS-12) did not fit the overall Rasch measurement model (χ^2^ 107.0; *p* < 0.001). Three items deviated significantly from the Rasch model (item fit residual beyond ± 2.5) with signs of dependency between item responses and disordered thresholds. Nevertheless, the PCS-12 was uni-dimensional with good internal consistency (person separation index [PSI] 0.83) and reasonable targeting. In contrast, the SF-12 Mental Component Score (MCS-12) had good overall model fit (χ^2^ 35.1; *p* = 0.07), reasonable targeting and good internal consistency (PSI 0.81).

**Conclusions:**

Rasch analysis suggests that there is general support for the reliability of the SF-12v2 as a measure of physical and mental health in people with lung cancer. However, the appropriateness of some items (e.g. pain) in the PCS-12 is questionable and further refinement of the scale including changing the response options may be required to improve the ability of the SF-12v2 to more appropriately assess the health status of this population.

**Supplementary Information:**

The online version contains supplementary material available at 10.1186/s12955-021-01794-w.

## Introduction

Lung cancer is one of the most commonly diagnosed cancers worldwide [1]. In the United States, it is estimated that 228,150 new cases will be diagnosed in 2019 [2]. In Australia, approximately 13,270 men and women will be newly diagnosed in 2019 which accounts for close to 9% of all cancers diagnosed [3]. Lung cancer is also the leading cause of cancer-related death and the biggest contributor to the overall burden of cancer [1, 4]. The advent of targeted and immune-directed therapies has improved survival for some patients, however, only 17% of those diagnosed are still alive five years after diagnosis [5]. In addition, those living with lung cancer report substantial physical and psychosocial distress associated with the disease and its treatment. There is therefore a need to understand the effects of patient morbidity including how patients with lung cancer perceive their health to impact on their physical and mental health status [6].

The routine use of patient-reported outcome measures (PROMs) allows health care providers to understand the patients’ perspective about the impact of treatments and care they have received, without interpretation from anyone else [7]. Patient-reported outcomes can contribute to person-centred care during both consultation and multidisciplinary team discussions [8]. The use of PROMs can also assist with monitoring outcomes of treatment (such as post-discharge complications or adverse events) and identifying patients at risk of problems or in need of specialist intervention [9]. Internationally, the healthcare environment is receptive to PROMs as a mechanism to incorporate patient perspectives in quality improvement, electronic data collections, value-based payments and shared decision making [8, 10]. By example, the National Health Service (NHS) in the United Kingdom introduced mandatory collection of PROMs for patients undergoing hip or knee replacement, hernia repair and varicose vein surgeries in 2009 [8]. In Australia, there is an emerging trend towards inclusion of PROMs within clinical quality registries such as for prostate cancer, percutaneous coronary interventions and heart failure [10]. The PROMs data collected in these registries are being used for performance monitoring, to support service improvement, and to inform future health policies [8].

The Victorian Lung Cancer Registry (VLCR) is a clinical quality registry that aims to capture all newly diagnosed lung cancer cases in participating public and private hospitals in Victoria, Australia [11]. The registry benchmarks hospital performance through a set of quality indicators that measure lung cancer care and outcomes, based on available literature and agreed upon by an expert committee. Between 2012 and 2016, the 12-item Short-Form Health Survey version 2 (SF-12v2) [12, 13] was used by VLCR to provide an indication of how a patient with lung cancer perceives their own health status. The SF-12v2 was used because with only 12 items, it has less respondent burden compared to the 36-item Short-Form Health Survey (SF-36) [14]. It also has a number of improvements over version 1 with changes to the response options for the role physical, role emotional, vitality and mental health items, and rewording of two items [15]. Australian population health data is available for both the Physical Component Summary (PCS-12) and Mental Component Summary (MCS-12) scores derived from the SF-12 [16, 17]. The SF-12 has been validated in both the general population and in a range of medical conditions [18–21]. In a sample of Americans with self-reported cancer, of which 2% had lung or bronchial cancer, the SF-12 was shown to have good internal consistency, and high convergent and predictive validity [18]. Nevertheless, no prior study has validated the use of SF-12v2 in patients with lung cancer within the Australian context.

Rasch analysis is a modern psychometric approach based on latent trait modelling that allows examination of key measurement and scaling properties of an outcome measure [22]. The Rasch measurement model is increasingly recognised as the gold standard for psychometric evaluations of outcome scales as it allows expected and observed results to be compared [23]. Previous studies examining the validity of the SF-12 using Rasch analysis in stroke and Parkinson’s disease identified issues with the measurement properties of both the PCS-12 and MCS-12 scores [24, 25]. No study has to date, used modern psychometric methods such as Rasch analysis, to assess the measurement properties of the SF-12v2 in patients with lung cancer. A previous study examining the validity of the SF-12 in patients with cancer used a classical test theory approach by correlating the derived summary measures with a similar instrument such as the EQ-5D [18]. The aim of this study was to extend the psychometric evaluations of the SF-12v2 within the Australian lung cancer population. In particular, we wanted to assess the structural validity of the SF-12v2 using Rasch analysis to ensure its validity and reliability in reflecting the health status of this population when used to benchmark patient outcomes by a clinical registry such as the VLCR.

## Methods

### Study population and participants

Data from all participants in the VLCR who completed the SF-12 between 2012 and 2016 were included in this study. Information from the VLCR is used to monitor the quality of care provided to patients newly diagnosed with primary lung cancer, including diagnosis and staging, treatment, and survival. The VLCR receives notification of patients discharged from participating health services with an International Classification Diseases (ICD) code for lung cancer, or suspected lung cancer (C34.0–C34.9, Z85.1, Z85.2). If a patient has a confirmed primary clinical or pathological diagnosis of lung cancer (excluding secondary lung cancers and mesothelioma) they are sent an explanatory statement and letter of invitation to participate in the registry [11]. A two-week window for consideration is provided and if during this period no request to ‘opt out consent’ is received then the patient is recruited to the VLCR and data collection commences. Registry governance is provided by a steering committee with representation from consumers, clinical and technical expert advisors and key stakeholders which oversee the registry activities, and supervise audit and monitoring of data collection and outcomes from each site. Ethical approval for this validation study was obtained from the Monash University Human Research Ethics Committee (MUHREC Project ID 13878).

### Data collection

Following consent, the VLCR collects an agreed minimum dataset from medical records, including sociodemographic and clinical data. Sociodemographic information included age, sex, country of birth, smoking status, past medical history (e.g. diabetes, renal insufficiency, respiratory conditions, myocardial infarction) and hospital type (i.e. public or private). Clinical data included cancer type (e.g. non-small cell lung cancer [NSCLC], small cell lung cancer [SCLC]), clinical and pathological (TNM) staging and lung cancer treatment (chemotherapy, radiotherapy, surgery). The Eastern Cooperative Oncology Group (ECOG) performance status scale was also collected as a clinician assessment of the patient’s ability to perform activities of daily living [26]. Between 2012 and 2016, vital status checks were made at 3, 6, 12, and 24 months following the date of diagnosis, and if participants were still alive, they were contacted by telephone to verify management details regarding their lung cancer before being asked to complete the SF-12v2 [11]. One interviewer was trained on how to collect data related to the management of lung cancer, as well as to administer the ECOG and SF-12v2 using an interview script that included both open- and closed-ended questions. Standard operating procedures were also developed to standardise the way in which the data were collected, and the same interviewer was used to contact the participants at each time point following diagnosis. On average, the telephone interview was completed within 15–20 min (5–10 min for management details and an additional 10 min for the ECOG and SF-12v2) and no issues were identified. Once obtained, all data were de-identified for further analyses.

### The SF-12 health survey

The SF-12v2 is an abbreviated version of the SF-36 [14] and the 12 items have been shown to predict at least 90% of the variance in the physical and mental summary scales derived from the SF-36 [21]. It is therefore an appropriate measure to capture the health status of patients when there are constraints on questionnaire length or when the focus is on patient-based assessments of physical and mental health [12]. In this study, the PCS-12 and MCS-12 scores, represented by six items each (Table 1), were computed and normalised for the SF-12v2 according to published algorithms [12]. Scores range from 0 to 100, with higher scores indicating better physical and mental health functioning [27]. A score of 50 or less on the PCS-12 has been recommended as a cut-off to determine a physical condition; while a score of 42 or less on the MCS-12 may be indicative of ‘clinical depression’ [27].

**Table 1:** The 12-item Short-Form Health Survey version 2 (SF-12v2) [12]

Scales	Item no	Contents	Response categories
Physical Component Summary (PCS-12)	1	General health	Excellent/very good/good/fair/poor
	2	Moderate activities	Limited a lot/limited a little/not limited at all
	3	Climb several flights of stairs	Limited a lot/limited a little/not limited at all
	4	Accomplished less (physical)	All of the time/most of the time/some of the time/a little of the time/none of the time
	5	Limited in kind of work	All of the time/most of the time/some of the time/a little of the time/none of the time
	8	Pain—interference	Not at all/a little bit/moderately/quite a bit/extremely
Mental Component Summary (MCS-12)	6	Accomplished less (emotional)	All of the time/most of the time/some of the time/a little of the time/none of the time
	7	Did work less carefully	All of the time/most of the time/some of the time/a little of the time/none of the time
	9	Calm and peaceful	All of the time/most of the time/some of the time/a little of the time/none of the time
	10	Energy or vitality	All of the time/most of the time/some of the time/a little of the time/none of the time
	11	Downhearted and blue	All of the time/most of the time/some of the time/a little of the time/none of the time
	12	Social limitations	All of the time/most of the time/some of the time/a little of the time/none of the time

### Statistical analysis

Descriptive statistics were used to summarise the sociodemographic and clinical characteristics of patients in the VLCR who completed the SF-12v2 three months following their lung cancer diagnosis. The PCS-12 and MCS-12 scores were analysed individually as two separate six-item physical and mental health scales in the Rasch analysis. Overall model fit, which includes overall fit, individual person fit and individual item fit, were assessed to determine whether the six items in the PCS-12 and MCS-12 met the expectations of the Rasch measurement model [23]. A non-significant value (*p* > 0.05) of the *χ*^2^ Item-Trait Interaction statistic indicated that the observed data fit the expectations of the Rasch model [22], while a residual standard deviation (SD) value of ≤ 1.5 in the item-person interaction statistics indicated satisfactory fit [22]. We are aware that the *χ*^2^ Item-Trait Interaction statistic is highly sensitive to sample size [28, 29]. Thus, a normed *χ*^2^ statistic value (i.e. *χ*^2^ divided by the degrees of freedom) of ≤ 2.5 was also used to indicate good model fit [28, 30, 31]. Finally, residual fit statistics of individual items and persons were inspected with values between ± 2.5 indicating adequate model fit [22].

To determine the structural validity of the SF-12v2, the following additional measurement properties were examined using Rasch analysis: (1) uni-dimensionality (including local dependency); (2) response thresholds; (3) internal consistency; (4) measurement invariance (item bias); and (5) targeting. The statistical tests and criteria used to assess these measurement properties are described in Table 2. All data were analysed using SPSS v25.0 (IBM Corporation, Armonk, New York). Rasch analysis was conducted using the RUMM2030 package with a partial credit model to allow thresholds to vary for each individual item (RUMM Laboratory Pty Ltd, Perth, Australia).

**Table 2:** Statistical tests and criteria for assessment of measurement properties of the SF-12v2

Measurement property	Definition	Statistical test and criteria for assessment
Dimensionality	Local dependency Response to an item should not be dependent on the response to another item Uni-dimensionality Extent to which items for the PCS-12 and MCS-12 measure one underlying construct	Person-item residual correlation values > 0.2 above the average correlation is indicative of local dependency [22, 23, 30] Uni-dimensionality confirmed if < 5% of significant t-tests between two most dissimilar subsets of items identified from the PCA of standardised residuals [22]. Where > 5% significant t-tests, uni-dimensionality supported if lower bounds of CI < 0.05 [22]
Response thresholds	Degree to which participants were able to discriminate between the response options for each item in the SF-12v**2**	Examination of pattern of thresholds from the threshold map Inspection of category probability curves. Thresholds considered to be ordered if each response option systematically has a point along the location continuum to be the most likely response
Internal consistency	The degree of inter-relatedness among SF-12v**2** items	Person separation index (PSI) values > 0.70 indicates good internal consistency (similar to Cronbach α values) [22]
Measurement invariance (item bias)	Whether or not different groups with similar characteristics (e.g. men vs women, public vs private) respond differently to a given item	Measured using differential item functioning (DIF). Uniform DIF is indicated by a significant main effect for the person factor (e.g. sex) using a Bonferroni adjusted *p* value for significance [22]. Non-uniform DIF is indicated by a significant interaction effect [22]
Targeting	Degree to which the PCS-12 and MCS-12 scores was targeted to patients with lung cancer	A well-targeted scale will have mean location logit score of zero [22]. Items will also be well-aligned on the person-item threshold distribution map

PCA, principal component analysis; CI, confidence interval; DIF, differential item functioning; PSI, person separation index; PCS-12, physical component summary; MC-12, mental component summary

## Results

### Participant characteristics

A total of 342 participants completed the SF-12v2 three months following their lung cancer diagnosis between 2012 and 2016. Over half of the participants were men (*n* = 191; 56%) with a mean age of 67 years (SD 11), which is reflective of the participants included in the VLCR [6]. The majority of participants (*n* = 288; 84%) presented with NSCLC and were actively treated for their cancer (*n* = 319; 93%). The most common treatment was surgical resections (*n* = 173; 51%) followed by chemotherapy (*n* = 168; 49%). Of those who had surgical resections, the most common resections were lobectomies (*n* = 114; 66%) and wedge resections (*n* = 24; 14%). The sociodemographic and clinical characteristics of the participants are described in Table 3.

**Table 3:** Characteristics of participants who completed the SF-12v2 between 2012 and 2016

	All participants (*n* = 342)
*Sociodemographic characteristics*	
Male, *n* (%)	191 (56)
Age, mean (SD)	67.3 (10.9)
Age group, *n* (%)	
< 70 years	190 (56)
≥ 70 years	152 (44)
Country of birth, *n* (%)	
Australia	241 (71)
Not Australia	91 (27)
Smoking status, *n* (%)	
Never smoked	28 (8)
Ex-smoker	211 (62)
Current smoker	83 (24)
Past medical history, *n* (%)	
Diabetes	53 (16)
Renal insufficiency (needing dialysis)	5 (2)
Myocardial infarction	66 (19)
Respiratory co-morbidity (FEV_1_ < 66%)	47 (14)
Neoplasm co-morbidity	76 (22)
Hospital type, *n* (%)	
Public	215 (63)
Private	127 (37)
*Clinical characteristics*	
Lung cancer type, *n* (%)	
Non-small cell lung cancer	288 (84)
Small cell lung cancer	35 (10)
Neuroendocrine	7 (2)
Other lung cancer	9 (3)
TNM staging, *n* (%)	
Non-small cell lung cancer	
Localised (I-II)	76 (26)
Locally advanced (III)	87 (30)
Metastatic (IV)	40 (14)
Unable to assess	85 (30)
Small cell lung cancer	
Extensive	20 (57)
Limited	12 (34)
Not stated	3 (9)
Active lung cancer treatment, *n* (%)	
Surgical resections	173 (51)
Chemotherapy	168 (49)
Radiotherapy	99 (29)
ECOG performance status, *n* (%)	
Independent (0–1)	206 (60)
Assistance (2–4)	136 (40)

SD, standard deviation FEV_1_, forced expiratory volume in one second; TNM staging, tumour, node and metastasis staging; ECOG, Eastern Cooperative Oncology Group

### Health status

The mean PCS-12 score for this sample of participants was 36.7 (SD 10.7; 95% CI 35.6, 37.9), which is considerably lower compared to Australian population health data (mean 45.3; 95% CI 45.3, 46.1) [16]. The mean MCS-12 score in this sample (mean 47.7; SD 10.4; 95% CI 46.6, 48.8) was reasonably well preserved and similar to the Australian general population mean (mean 52.1; 95% CI 51.8, 52.4) [17]. Of note, 299 participants (87%) recorded a score of 50 or less on the PCS-12 indicating they had a physical condition whilst only 105 participants (31%) scored less than 42 (indicating they have clinical depression) on the MCS-12.

### Structural validity of the PCS-12

Analysis of the PCS-12 showed a lack of fit to the overall Rasch measurement model with a significant χ^2^ Item-Trait Interaction statistic and a normed χ^2^ statistic value of 5.94 (Table 4). A degree of item misfit was also observed (fit residual mean − 0.50; SD 3.36) and analysis of individual item fit statistics indicated that three items deviated significantly from the Rasch model (Additional file 1). Items 4 (‘*accomplished less than you would like as a result of your physical health’*) and 5 (‘*were limited in the kind of work as a result of your physical health’*) had fit residual values that were less than − 2.5, which suggests potential item redundancy. In contrast, item 8 (‘*how much did pain interfere with your normal work’*) had a fit residual value that was greater than 2.5 which suggests that it may not be measuring the same underlying construct as the other items in the PCS-12. Although no serious person misfit was observed (fit residual mean − 0.46; SD 1.19), analysis of individual person statistics indicated that three participants had positive fit residual values greater than 2.5. Inspection of person-by-item responses showed that unexpected responses were observed for item 8, as well as items 2 (‘*moderate activities’)* and 3 (‘*climbing several flights of stairs’*). Participants appeared to have misunderstood or responded inappropriately to these items.

**Table 4:** Overall Rasch model fit statistics and reliability of the SF-12v2^a^

	Ideal	PCS-12	PCS-12 (subtest analyses)^d^	MCS-12
*Total item-trait interaction*				
Total item χ^2^		107.0	38.1	35.1
d*f*		18	12	24
*p* value	> 0.05	< 0.001	< 0.001	0.067
Normed χ^2 b^	≤ 2.5	5.94	3.18	1.5
*Items*				
Fit residual (mean)	0	-0.50	0.05	0.10
Fit residual (SD)	< 1.5	3.36	2.07	1.15
*Persons*				
Fit residual (mean)	0	-0.46	-0.38	-0.34
Fit residual (SD)	< 1.5	1.19	0.94	1.09
*Uni-dimensionality*				
Equating *t*-tests Binomial dimensionality test (95% CI) Person-item residual correlation	< 0.05 (lower limit < 0.05) < 0.2	0.06 (0.03, 0.08) > 0.2 for items 2 and 3 > 0.2 for items 4 and 5	0.04 – < 0.2 for all items	0.02 – < 0.2 for all items
*Person separation index*^c^	> 0.7	0.83	0.72	0.81
Equivalent Cronbach’s α	> 0.7	0.85	0.73	0.83

^a^As analysed using RUMM2030 (Rumm Laboratory Pty Ltd., Perth) for Windows

PCS-12, physical component summary; MCS-12, mental component summary; SD, standard deviation; CI, confidence interval; d*f*, degrees of freedom

^b^Ratio of χ^2^ value to degrees of freedom

^c^Rasch based reliability statistic (analogous to Cronbach’s α)

^d^Subtest analyses for PCS-12 combining items 2 and 3 as well as items 4 and 5

#### Uni-dimensionality

Local dependency was observed between items 2 and 3, as well as items 4 and 5 with person-item residual correlations of 0.50 and 0.77 respectively, which was > 0.2 above the average correlation $({\overline{Q} }_{3})$ of − 0.13 [32]. Despite this, we found some evidence to support uni-dimensionality of the PCS-12. Although t-tests between the two most dissimilar subsets of items identified from the PCA of standardised residuals was > 5% (Table 4), the lower bound of the 95% CI included 0.05 indicating that all six items measured the same underlying construct of physical health.

#### Response thresholds

**Fig. 1:**
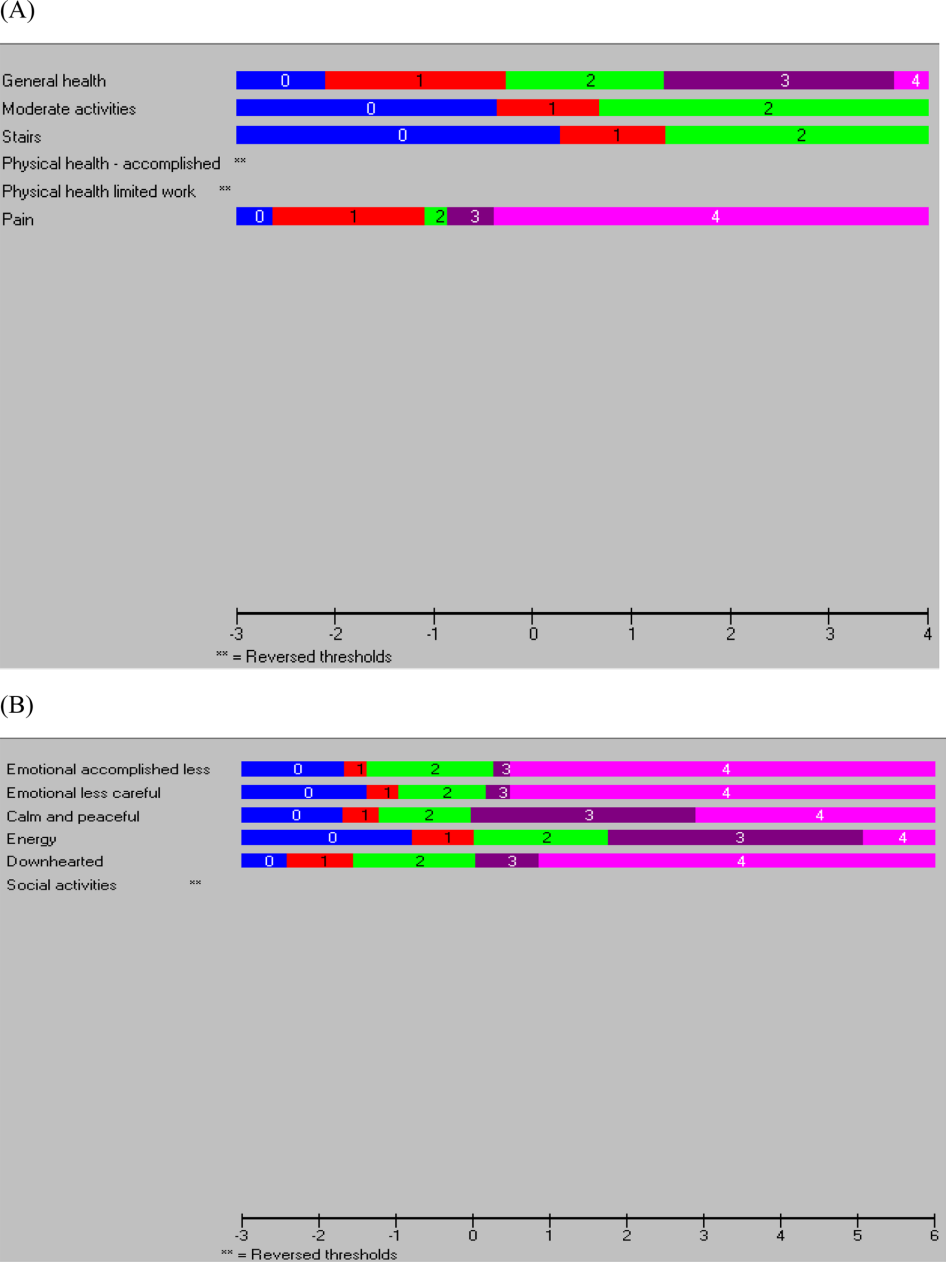
Response thresholds for the six items in the (**a**) PCS-12 and (**b**) MCS-12

Disordered thresholds were observed for items 4 and 5 (Fig. 1a), and inspection of the category probability curves indicated that participants were not using the 5-point rating scale (‘*all of the time*’ to ‘*none of the time*’) in a consistent manner (Additional file 2). There was a greater probability that they would choose the categories on either side of ‘*a little of the time*’. Participants also appeared to have difficulty distinguishing between the different options of the 5-point rating scale for item 8 that ranged from ‘*not at all*’ to ‘*extremely*’. They were more likely to choose the categories to either side of ‘*quite a bit*’ and ‘*moderately*’.

#### Internal consistency

The person separation index (PSI) statistic for the PCS-12 was 0.83, indicating good internal consistency reliability. It is important to note that this value was not artificially inflated by the correlation observed between items 2 and 3 as well as items 4 and 5 as subtest analyses showed that the PSI value did not drop below the 0.7 threshold (Table 4 and Additional file 3).

#### Measurement invariance (item bias)

Statistical tests of differential item functioning (DIF) was used to determine whether participants responded differently to each item of the PCS-12 according to their age group (< 70 years vs ≥ 70 years), sex (male vs female), hospital type (public vs private) and ECOG functional level (independent vs assistance). Violation of measurement invariance, specifically uniform DIF where participants responded differently in a consistent manner, was observed for item 8 with respect to their age group and ECOG functional level. We were also interested in whether participants with NSCLC at different TNM stage would respond differently to the six items. However, no significant DIF was observed for the PCS-12 items indicating that this characteristic did not influence participants’ response to the items.

#### Targeting

**Fig. 2:**
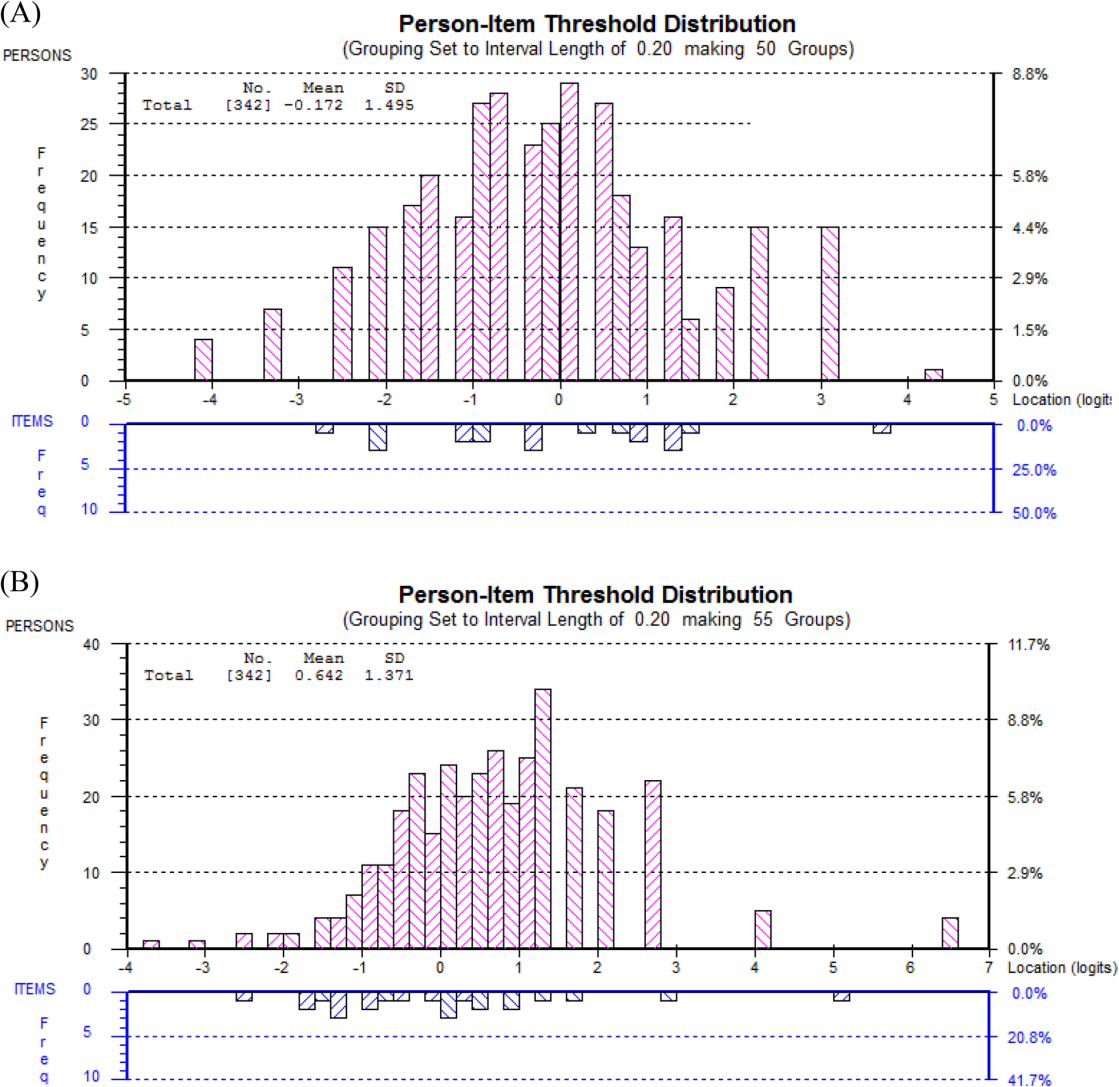
Person-item threshold distribution depicting targeting for **a** PCS-12 and **b** MCS-12. Distributions of the locations of people (upper panel) and items (lower panel) on the common logit metric (negative values = poor health; positive values = better health)

The PCS-12 displayed reasonable targeting (Fig. 2a) with a mean logit score of − 0.17, although there was a clustering of participants with moderate physical health status and no corresponding scale item.

### Structural validity of the MCS-12

As shown in Table 4, the MCS-12 met the expectations of the overall Rasch measurement model for good overall model fit (χ^2^ Item-Trait Interaction statistic *p* = 0.07 and normed χ^2^ statistic value of 1.46). Inspection of individual item-fit and person-fit statistics also indicated that there were no mis-fitting items or persons with all fit residual values between ± 2.5 (Additional File 1).

#### Uni-dimensionality

All six items of the MCS-12 demonstrated uni-dimensionality, with no local dependency observed.

#### Response thresholds

Ordered thresholds were observed for all items except item 12 (‘*has physical health or emotional problems interfered with social activities’*) (Fig. 1b). Further inspection of category probability curves, however, indicated that participants were not using the 5-point rating scale (‘*all of the time*’ to ‘*none of the time*’) in a consistent manner (Additional file 2) for this item as well as for items 6 (‘*accomplished less due to emotional problems’*) and 7 (‘*did work less carefully due to emotional problems’*). In particular, participants appeared to have a greater probability of choosing the categories to either side of ‘*most of the time*’ and ‘*a little of the time*’.

#### Internal consistency

The MCS-12 displayed good internal consistency reliability with a PSI of 0.81 and an equivalent Cronbach’s α of 0.83.

#### Measurement invariance (item bias)

Measurement invariance was not evident for the MCS-12 with respect to age group and sex. No item bias was also evident amongst participants with NSCLC at different TNM stages. However, uniform DIF (*p* < 0.05) was observed for item 9 (‘*felt calm and peaceful’*) between public and private patients, as well as for item 12 between those who were independent or required assistance based on the ECOG scale of performance status.

#### Targeting

The MCS-12 was reasonably well-targeted (Fig. 2b) with a mean logit score of − 0.64, although there may be a slight ceiling effect with insufficient items assessing individuals at the higher end of the mental health spectrum.

## Discussion

This study has provided new information regarding the structural validity of the SF-12v2 as a measure of physical and mental health status in patients with a recent diagnosis of lung cancer enrolled in the VLCR. We found evidence to support the use of the SF-12v2, in particular the MCS-12, to assess aspects of mental health in this population. All six items of the PCS-12 and MCS-12 demonstrated uni-dimensionality, which is a critical property of good measurement tools [23, 33]. However, we did identify some issues with the six items that make up the PCS-12, which may limit its ability to precisely measure the physical health status of patients with lung cancer.

The findings of this study are consistent with previous studies using Rasch analysis to examine the structural validity of the SF-12 in people with Parkinson’s disease (PD) and stroke [24, 25]. These studies identified issues with overall model fit for the PCS-12, as well as local response dependencies for items 4 and 5 [24, 25]. Participants responded to both items in the same manner which is not surprising given the similarity in the item contents (Table 1). Whilst explorative deletion of item 5 appeared to improve model fit in people with PD [24], it may not be practical or feasible to use different versions of the SF-12v2 in different health conditions. One of the advantages of the SF-12v2 is that it allows the health status of people with lung cancer to be compared with healthy individuals (e.g. Australian population health data) or those with other medical conditions [34]. Utility values (SF-6D) can also be derived from the SF-12v2 which can be used to determine quality-adjusted life years (QALYs) [35]. If items were to be deleted from the SF-12v2, its use as a generic measure of health status and quality of life may be compromised. Thus, further studies in larger samples are needed to determine model fit and measurement precision will likely improve if items are thereby adapted.

In contrast to previous studies, we did not observe any item misfit or local dependency for the six MCS-12 items [24, 25]. In our sample of patients with lung cancer, the MCS-12 displayed overall fit to the Rasch model, good internal consistency reliability and was reasonably well-targeted. The items that make up the MCS-12 appear to be able to appropriately measure emotional and affective problems in this population. There were, however, some indications that participants were not using the 5-point rating scale (‘*all of the time*’ to ‘*none of the time*’) in a consistent manner for several items (items 6, 7 and 12). It is worth noting that this disordering was relatively minor and other items using the same response options did not display disordered thresholds. As such, further studies are warranted before we can confirm whether there is a need to modify the response categories of these items.

We found that all items of the SF-12v2 worked consistently among men and women with lung cancer as well as those with NSCLC at different TNM stages. Minor bias was evident for items 8, 9 and 12 according to age, hospital type and level of functional status. This means that care needs to be taken if we wish to compare the physical and mental health status of patients with lung cancer across these sociodemographic and clinical subgroups [36]. We do need to acknowledge that the PCS-12 and MCS-12 scores in this study were derived using the standard scoring algorithm which has been shown to yield ambiguous and misleading results as it assumes that there is no association between physical and mental health [37, 38]. Simulation data indicates that good physical health scores may reduce mental health scores and vice versa [37]. This may explain the relatively low PCS-12 scores observed in our sample despite most patients being independent according to the ECOG performance status scale. Given that the SF-12v2 only generates summary scores, this can make it difficult to identify any potential problems caused by the standard scoring algorithm [38]. Thus, future studies may need to consider using alternative scoring procedures, such as the RAND-12 Health Status Inventory (HSI) [39], which may provide more valid representations of physical and mental health because it employs Rasch-based item scoring [37]. The use of country-specific weights to derive the summary scores should also be considered in order to improve the measurement properties of the SF-12v2 [38].

It is also important to consider the potential need for a PROM that is specific to our population of interest i.e. patients with lung cancer. Whilst a generic measure such as the SF-12v2 is useful as it allows comparisons across different health conditions and the ability to undertake economic studies, a lung cancer disease-specific measure such as the European Organisation for Research and Treatment of Cancer Quality of Life Questionnaire—Lung Cancer module (EORTC QLQ-LC13) [40] or the Functional Assessment of Cancer Therapy – Lung (FACT-L) [41] will allow us to capture the specific quality of life issues that may be pertinent to this population. Additionally, the SF-12v2 was derived from the SF-36 where items were selected by the authors based on the Medical Outcomes Study [14]. Patients did not appear to be involved in the identification of domains, outcomes or item wording for both the SF-36 and SF-12v2. Given that patients and health care professionals rank the importance of health outcomes differently [42], a PROM derived using genuine patient input that can be administered within a clinical quality registry such as the VLCR may be warranted [43]. Consideration also needs to be given to the growing use of computer adaptive testing to tailor the inclusion of items in PROMs, which is the approach used by the Patient-Reported Outcomes Measurement Information System (PROMIS®) [44]. Whilst the widespread application and short-form nature of the SF-12v2 may make it attractive for potential users, it is a legacy instrument (together with the SF-36) and may have limited applicability in clinical quality registries particularly if the standard scoring algorithm is used [37, 45].

A key strength of this study is the use of Rasch analysis, which has been recognised as the gold standard for the psychometric evaluations of outcome scales [33, 46]. Findings from this study can therefore be used to inform the refinement of the SF-12v2 such as removing misfitting items or modifying response categories to improve its measurement properties. However, some limitations need to be taken into consideration. Firstly, our sample size (*n* = 342) may have contributed to the significant *χ*^2^ probability values observed for the PCS-12 as small deviations from model fit will be statistically significant with sufficiently large sample sizes [29]. In addition, we only included participants who were recently diagnosed 3-months following a definitive diagnosis. This may limit the generalisability of our findings. The TNM staging data was also unavailable for many of the patients with NSCLC. This data field was poorly completed during the initial establishment years of the registry, although the proportion of missing data (30%) is consistent with data published by the Victorian Cancer Council [47]. The mode of administration of the SF-12v2 (i.e. via telephone) may have affected the way in which participants recalled the response options to each item leading to the observed issues with response thresholds. Finally, we are unable to evaluate whether the measurement properties of the SF-12 would change over time as we only included data from one time point for this set of analyses.

## Conclusion

This study has provided important insights into the measurement properties and structural validity of the SF-12v2. We found general support for the reliability of the SF-12v2 as a measure of physical and mental health in people with lung cancer. However, the appropriateness of some items (e.g. pain) in the PCS-12 is questionable and further refinement of the scale including changing the response options may be required to improve the ability of the SF-12v2 to more appropriately assess the health status of this population. Until such evidence is available, caution is required when using the SF-12v2 as an outcome measure in people with lung cancer.

## Supplementary Information

**Additional file 1**. Rasch item and fit statistics for the SF-12v2.

**Additional file 2**. Category probability curves for items 4, 5, 6, 7, 8 and 12 of the SF-12v2.

**Additional file 3**. Rasch item and fit statistics for subtest analyses of the PCS-12.

## Data Availability

Data cannot be shared publicly because we did not seek approval from study participants to have data shared publicly. Data are available from the Monash University Human Research Ethics Committee (contact via the Executive Officer at Monash University: muhrec@monash.edu) for researchers who meet the criteria for access to confidential data.

## Abbreviations

CI: Confidence interval
DIF: Differential item functioning
ECOG: Eastern Cooperative Oncology Group
HIS: Health Services Inventory
ICD: International Classification of Diseases
MCS-12: SF-12 Mental Component Score
NHS: National Health Service
NSCLC: Non-small cell lung cancer
PCA: Principal component analysis
PCS-12: SF-12 Physical Component Score
PD: Parkinson’s disease
PROM: Patient reported outcome measure
PSI: Person separation index
QALY: Quality adjusted life years
SCLC: Small cell lung cancer
SD: Standard deviation
SF-12v2: 12-Item Short-Form Health Survey version 2
SF-36: 36-Item Short-Form Health Survey
TNM: Tumour, node and metastases staging
VLCR: Victorian Lung Cancer Registry
