# Supplementary material for: Measurement properties of the 12-item Short Form Health Survey version 2 in Australians with lung cancer: a Rasch analysis
Source: Health Qual Life Outcomes. 2021 May 31;19:157. doi: 10.1186/s12955-021-01794-w (PMC8165769; doi:10.1186/s12955-021-01794-w)
Supplement: Supplementary file 1 — Additional file 1. Rasch item and fit statistics for the SF-12v2. [file 12955_2021_1794_MOESM1_ESM.docx]

Additional File 1: Rasch item and fit statistics for the SF-12v2

|  | Item | Item  scores | Item  statistics^ϯ^ | Fit statistics | | | | | |
| --- | --- | --- | --- | --- | --- | --- | --- | --- | --- |
|  |  | Mean  (SD) | Location (SE) | Residuals* | χ^2§^ | d*f* | *p*-value^‡^ | F statistic^∞^ | *p*-value^‡^ |
| PCS-12 | 1 | 2.58 (0.99) | 0.66 (0.08) | 0.96 | 7.16 | 3 | 0.067 | 2.43 | 0.065 |
|  | 2 | 1.85 (0.82) | 0.16 (0.09) | -0.09 | 3.61 | 3 | 0.307 | 1.69 | 0.169 |
|  | 3 | 1.63 (0.77) | 0.81 (0.10) | -0.65 | 4.95 | 3 | 0.176 | 2.28 | 0.080 |
|  | 4 | 2.92 (1.24) | -0.20 (0.07) | **-3.59** | 15.76 | 3 | 0.001 | 10.96 | <0.001 |
|  | 5 | 2.93 (1.22) | -0.19 (0.07) | **-4.49** | 19.38 | 3 | <0.001 | 15.26 | <0.001 |
|  | 8 | 3.84 (1.25) | -1.24 (0.06) | **4.86** | 56.10 | 3 | <0.001 | 11.49 | <0.001 |
| MCS-12 | 6 | 3.89 (1.14) | -0.56 (0.07) | -0.94 | 8.19 | 4 | 0.085 | 2.32 | 0.057 |
|  | 7 | 3.82 (1.19) | -0.41 (0.06) | 0.56 | 4.69 | 4 | 0.321 | 1.26 | 0.287 |
|  | 9 | 3.53 (0.97) | -0.00 (0.08) | -0.49 | 4.06 | 4 | 0.398 | 1.12 | 0.345 |
|  | 10 | 2.70 (1.01) | 1.53 (0.07) | 2.00 | 5.30 | 4 | 0.258 | 1.09 | 0.260 |
|  | 11 | 3.91 (1.04) | -0.76 (0.07) | -1.00 | 8.86 | 4 | 0.065 | 2.78 | 0.027 |
|  | 12 | 3.28 (1.30) | 0.21 (0.06) | 0.46 | 3.99 | 4 | 0.408 | 1.14 | 0.340 |

^ϯ^Expressed in linear log-odds units (logits), with mean item location set at 0 for each scale.

*Log residuals summarise the deviation of observed from expected responses. Deviation from the recommended range of +2.5, indicating item misfit, are in bold typeface [22].

^§^χ^2^ values summarise the deviation of observed from expected responses across the sample. Higher absolute χ^2^ values represent larger deviations.

^∞^One way ANOVAs of deviations from model expectation across the sample.

^‡^Bonferroni corrected p-value threshold is 0.0083
