# Supplementary figures and images for: Measurement properties of the 12-item Short Form Health Survey version 2 in Australians with lung cancer: a Rasch analysis
Source: Health Qual Life Outcomes. 2021 May 31;19:157. doi: 10.1186/s12955-021-01794-w (PMC8165769; doi:10.1186/s12955-021-01794-w)

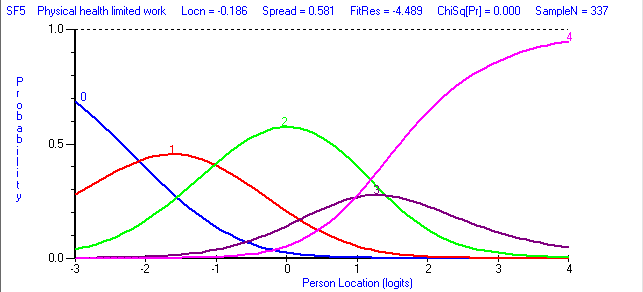

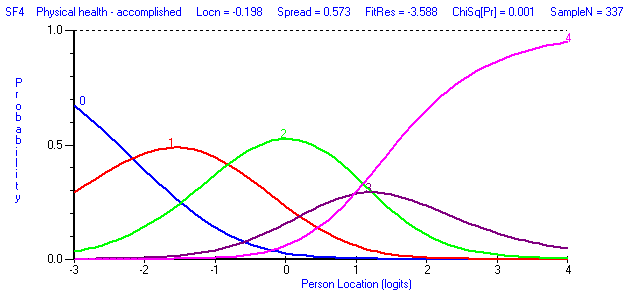


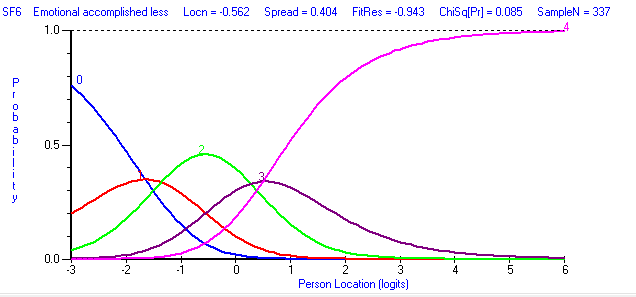


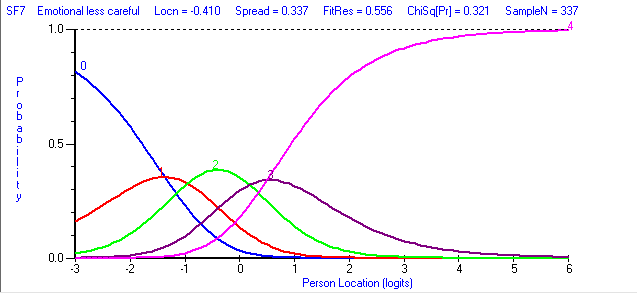


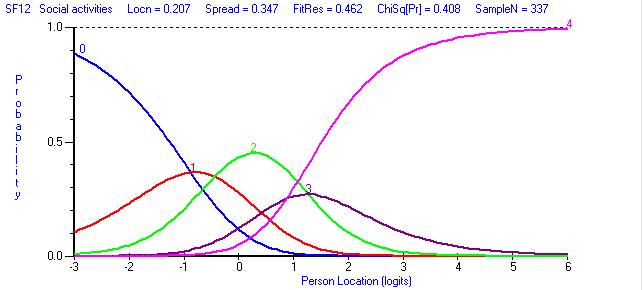

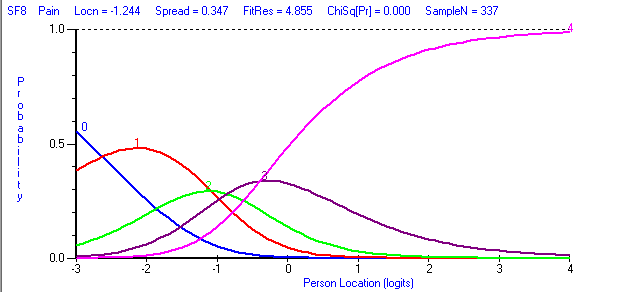


Additional File 2: Category probability curves for items 4, 5, 6, 7, 8 and 12 of the SF-12v2

Supplement: Supplementary file 2 — Additional file 2. Category probability curves for items 4, 5, 6, 7, 8 and 12 of the SF-12v2. [file 12955_2021_1794_MOESM2_ESM.docx]
