# Supplementary material for: Measurement properties of the 12-item Short Form Health Survey version 2 in Australians with lung cancer: a Rasch analysis
Source: Health Qual Life Outcomes. 2021 May 31;19:157. doi: 10.1186/s12955-021-01794-w (PMC8165769; doi:10.1186/s12955-021-01794-w)
Supplement: Supplementary file 3 — Additional file 3. Rasch item and fit statistics for subtest analyses of the PCS-12. [file 12955_2021_1794_MOESM3_ESM.docx]

Additional File 3: Rasch item and fit statistics for subtest analyses of the PCS-12

| Item | Item  scores | Item  statistics^ϯ^ | Fit statistics | | | | | |
| --- | --- | --- | --- | --- | --- | --- | --- | --- |
|  | Mean  (SD) | Location (SE) | Residuals* | χ^2§^ | d*f* | *p*-value^‡^ | F statistic^∞^ | *p*-value^‡^ |
| 1 | 2.58 (0.99) | 0.39 (0.05) | 0.21 | 1.41 | 3 | 0.704 | 0.95 | 0.417 |
| 2+3 | 1.85 (0.82) | -0.04 (0.04) | **-2.59** | 10.91 | 3 | 0.012 | 8.28 | <0.001 |
| 4+5 | 1.63 (0.77) | 0.61 (0.07) | 0.08 | 14.66 | 3 | 0.002 | 6.69 | <0.001 |
| 8 | 3.84 (1.25) | -0.96 (0.06) | 2.46 | 11.56 | 3 | 0.011 | 3.03 | 0.030 |

^ϯ^Expressed in linear log-odds units (logits), with mean item location set at 0 for each scale.

*Log residuals summarise the deviation of observed from expected responses. Deviation from the recommended range of +2.5, indicating item misfit, are in bold typeface [22].

^§^χ^2^ values summarise the deviation of observed from expected responses across the sample. Higher absolute χ^2^ values represent larger deviations.

^∞^One way ANOVAs of deviations from model expectation across the sample.

^‡^Bonferroni corrected p-value threshold is 0.0083
